# Effects of excessive alcohol drinking on nicotine biotransformation in rats

**DOI:** 10.1038/s41598-022-15199-2

**Published:** 2022-06-30

**Authors:** Joanna Kasprzyk, Wojciech Piekoszewski, Artur Tezyk, Maksymilian Kulza, Ewa Florek

**Affiliations:** 1 Laboratory of High Resolution Mass Spectrometry, Faculty of Chemistry, Jagiellonian University, Krakow, Poland; 2 Department of Analytical Chemistry, Faculty of Chemistry, Jagiellonian University, Krakow, Poland; 3 School of Biomedicine Far Eastern, Federal University, Vladivostok, Russian Federation; 4 Department of Forensic Medicine, Poznan University of Medical Science, Poznan, Poland; 5 Laboratory of Environmental Research, Department of Toxicology, Poznan University of Medical Sciences, 30 Dojazd Str, 60-631 Poznan, Poland

**Keywords:** Chemical biology, Biomarkers

## Abstract

Alcohol and nicotine (tobacco smoke) are often used together, and taking both addictive substances is associated with an increased risk of certain diseases. It is extremely important to understand the pharmacodynamic and pharmacokinetic mechanisms of the interaction between nicotine and ethanol, which are still not fully understood. The study aimed to evaluate the influence of chronic alcohol consumption on nicotine biotransformation in ethanol-preferring and non-preferring male and female rats. Rats were divided into four groups depending on their alcohol preferences and gender. Nicotine, nornicotine, nicotine N-oxide, cotinine, trans-3'-hydroxycotinine, and cotinine N-oxide in rats plasma were determined by LC–MS/MS after five days of exposure to tobacco smoke. A non-compartmental analysis of nicotine and its metabolites was used for pharmacokinetic parameters calculation. Our experimental results showed that the rate of nicotine elimination depends on gender, regardless of alcohol preferences (significantly slower in females than in males). Mean residence timeof nornicotine, cotinine, and trans-3'-hydroxycotinine were significantly higher in alcohol-preferring male rats than in alcohol preferring female rats. In non-alcohol preferring female rats compared to ethanol-preferring female rats, significantly more nicotine N-oxide (fivefold) and trans-3'-hydroxycotinine (twofold) reached the general circulation unchanged. Drinking ethanol influenced the elimination of nornicotine and cotinine in male rats. Ethanol consumption was identified as a modifier of nicotine pharmacokinetics and this was gender-dependent.

## Introduction

Alcohol and tobacco are often used together and are of interest to scientists, clinicians and sociologists in the context of a serious medical, sociological, and economic problem on a global scale. Some authors have shown a positive correlation between the amount of nicotine absorbed from smoke and ethanol consumption, as well as between the risk of being a heavy smoker and the degree of ethanol dependence^1,2^. A common effect of exposure to tobacco smoke, and alcohol drinking is oxidative stress and damage to proteins, polysaccharides, lipids, and DNA, leading to the intensification of pro-inflammatory reactions, accelerated aging, impaired immunity, and the development of neoplastic diseases^3,4^. Despite extensive research, the mechanisms of interaction between these drugs of abuse (xenobiotics) are still not fully understood.

**Figure 1:**
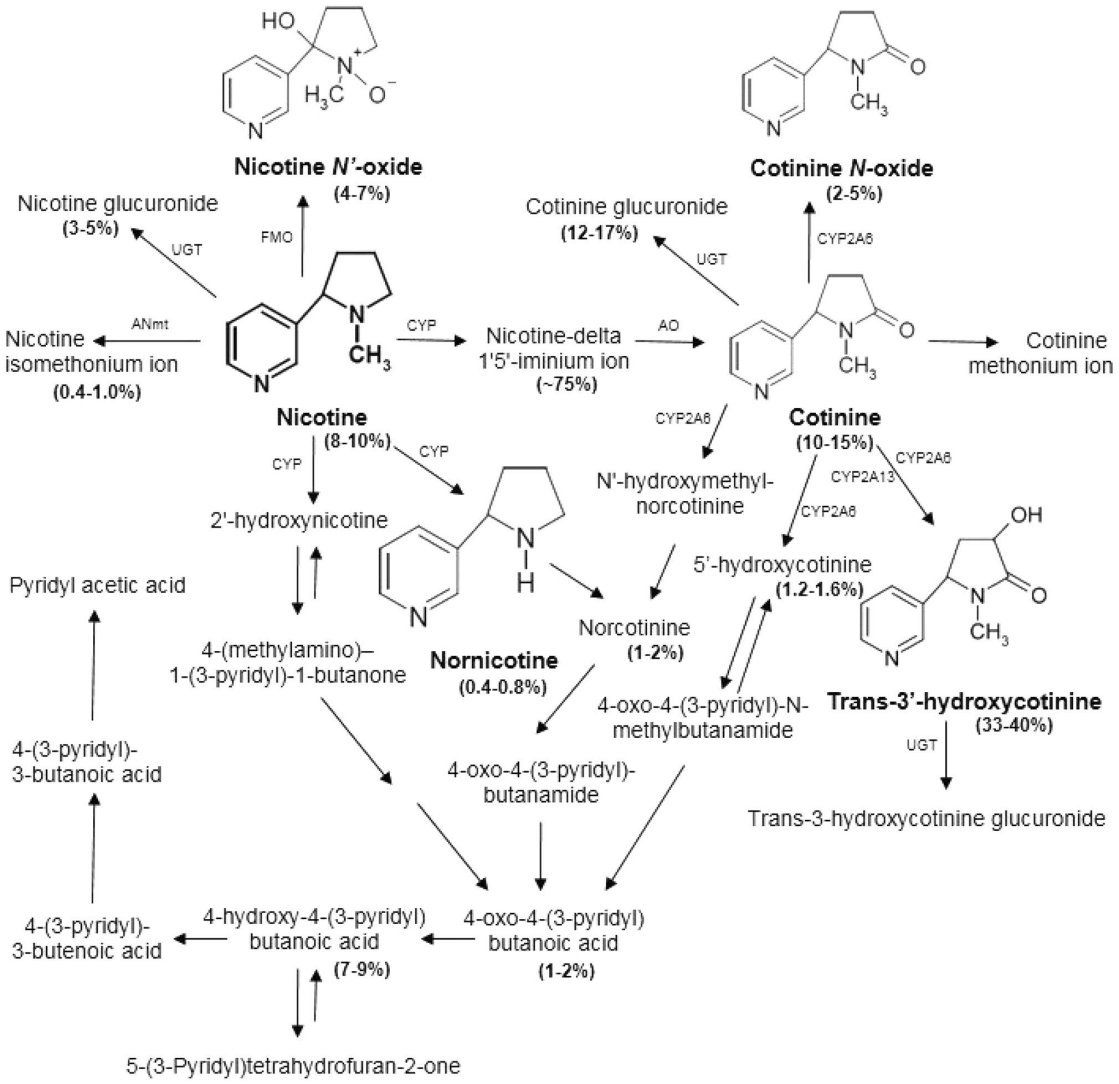
A scheme of quantitative nicotine metabolism, based on estimates of average excretion of metabolites as a percent of total human urinary nicotine. (%)—the percentage excreted in the urine of nicotine and its metabolites; CYP—cytochrome P450 enzymes; UGT—UDP-glucuronosyltransferase; FMO—flavin-containing monooxygenase; AO—aldehyde oxidase; ANmt—amine N-methyltransferase. A sheme based on publication data^19,26,27^.

Alcohol and nicotine can affect people differently depending on gender^5,6^, age^7–9^, diet, body mass index (BMI)^10^, genetic factors^11^ biological factors (estrogen levels), and environmental factors (alcohol consumption) that have an effect on the activity of the enzyme CYP2A6^12^. During smoking, nicotine (NIC), the main addictive ingredient of tobacco smoke, is absorbed 82–92% by the lungs in a pH-dependent manner^13^. Absorption can also occur through the mouth, skin, and gastrointestinal tract^14,15^. Nicotine absorbed into the pulmonary circulation is rapidly distributed into such tissues as the brain and heart. Plasma NIC levels increase within 12 min after cigarette smoking and slowly decrease over the next few hours^16^. Nicotine reaches the central nervous system within 20 s of inhaling tobacco smoke, and produces the intense “positive” pharmacological effect. NIC is rapidly metabolized and the elimination half-life is within the range 1–4 h, averaging 2 h^17,18^. The major pathway of NIC metabolism (in humans) is the oxidation of carbon to cotinine (COT) by CYP2A6 and to a lesser extent by CYP2B6, CYP2D6, CYP2E1, and by glucuronidation, catalyzed by UDP-glucuronosyl transferases (UGT), or oxidation by flavin monooxygenases (FMO; see Fig. 1). In addition to cotinine, about 20 derivatives are formed as a result of the biotransformation of NIC, mainly nornicotine (NOR) (0.4–0.8%), nicotine-N-oxide (NICNO) (4–7%), trans-3'-hydroxycotine (3HC) (33–40%), and cotinine-N-oxide (COTNO) (2–5%) (see Fig. 1). CYP2A6 is also involved in the metabolism of cotinine to trans 3'-hydroxycotinine. This isozyme presents low activity in rodents, therefore rodent COT may show a different metabolism compared to human COT^19^. In rodents, NIC is mainly metabolized by CYP2B1 and CYP2B2 2^20^. Information on the participation of CYP450 in nicotine metabolism in rats is rare, research on this topic was conducted, among others, by Nakajama et al. It showed that CYP2B1 is the major contributor to the metabolism of nicotine, but not the biotransformation of NIC by CYP2C2 and 3A2, and the contribution of CYP1A1, 2A1, 2A2, 2C7, 2C12, 2C13, 2EI and 4A1 to nicotine oxidation was not shown^21^. The lower CYP2A6 activity results in slower metabolism of COT to 3HC compared to the rate of cotinine formation from nicotine^22^. The half-life of COT is approximately 13–19 h, which is much longer than that of NIC or 3HC (approximately 5 h)^23,24^. A small fraction of nicotine is metabolized to nornicotine (NOR)^13^, which may contribute to the behavioral and enhancing effects of nicotine addiction^25^.

The second major compound used in research (ethanol) in humans, is metabolized mainly by alcohol dehydrogenase (ADH). Some studies suggest that, in addition, CYP2E1 very likely metabolizes 20–60% of ethanol depending on blood alcohol concentration, and drinking frequency^27^.

Subchronic ethanol dosing induces hepatic nicotine-metabolizing CYP2B1, and subchronic administration of nicotine induces CYP2E1 in rats^27^. Exposure to ethanol and nicotine, alone or in combination, can modify the pharmacokinetics of nicotine^28^. The aim of this study was to evaluate the effect of chronic alcohol administration on the biotransformation of nicotine in an animal model.

## Material and methods

### Animals

The study design was approved by the Ethics Committee for Animal Experiments in Poznan, Poland. The study is reported in accordance with ARRIVE guidelines. All procedures concerning the handling and use of laboratory animals were performed in accordance with European Union (UE) regulations under Directive 2010/63/EU on the protection of animals used for scientific purposes. Experiments were carried out in accordance with the so-called 3Rs principle (Replacement, Reduction, Refinement) to protect animals. In order to obtain consistent data, the study was based on the required minimum number of animals and observation time.

White, male and female Wistar rats, aged three months (average body weight: 361 g male and 189 g female), born and reared in the Animal House of the Department of Toxicology, at the Poznan University of Medical Sciences, Poznan, Poland, were housed in polypropylene cages (33 × 17.8 × 40 cm) (n = 1 rat/cage) with autoclaved pine sawdust litter under controlled environmental conditions (12 h light/dark cycle: 7 am–7 pm; temperature: 22 ± 2 °C; air humidity: 50–60%). The animals were allowed to acclimatize for two weeks before beginning the experiment (Department of Toxicology) with ad libitum access to water and wholesome feed. The water was sterilized before being given to the animals. The animals were fed Labofeed B Plant ("Morawski" Feed Production Plant—the dietary formula was created based on the recommendations of the National Research Council in the field of Nutrient Requirements of Laboratory Animals) and were weighed every 2 or 3 days for alcohol dose adjustment.

**Figure 2:**
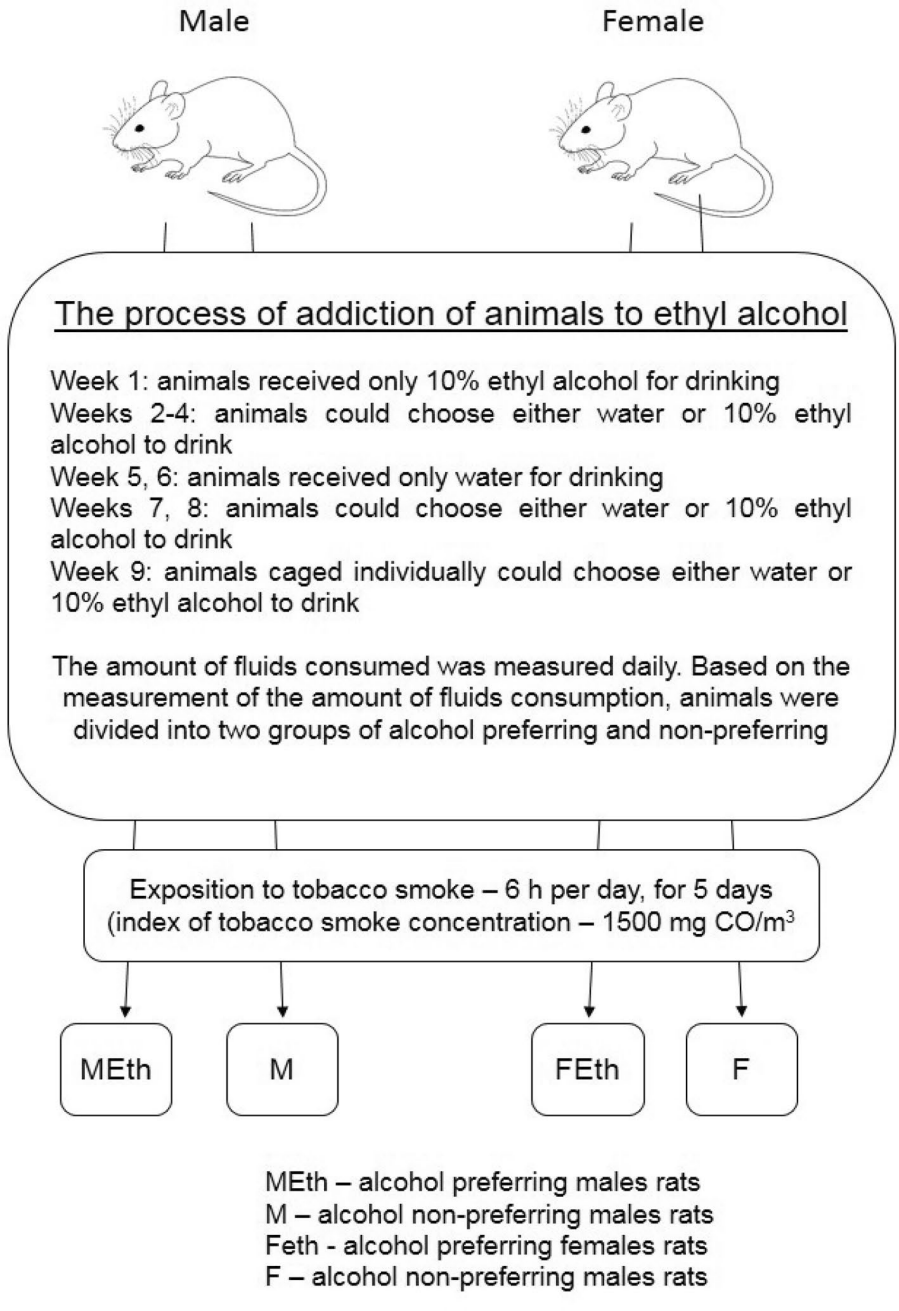
The schedule of the experiment.

The number of animals used in r the next step of the experiment was selected from this group. Selection alcohol-preferring and non-alcohol preferring rats lasted 9 weeks according to the previously established protocol^29–32^. Then the necessary number of rats was selected—thirty-six female and thirty-six male rats were used for the experiment half of the male (MEth) and female rats (FEth) preferred alcohol, and half of the male rats (M); and female rats (F) did not prefer alcohol. The schedule of the experiment is presented in Fig. 2.

At the end of the exposure, the maximum concentration of ethyl alcohol (in ethanol preferring rats) was observed after 0.25 h and ranged averaged 1.47 mg/L for females and 1.58 mg/L for males (unpublished data). Then, the animals were placed in the toxicological chamber^33^.

The product used to expose animals to tobacco smoke was cigarettes without a filter, made by Imperial Tobacco Polska S.A. The content of selected components of tobacco smoke selected for the experimental cigarettes was 10 mg of tar, 0.9 mg of nicotine, and 8 mg of carbon monoxide. The tobacco products were placed in a combustion scrubber while controlling the content of carbon monoxide (CO) in the air of the chamber (CO sensor with patented auto-calibration procedure). The content of carbon monoxide in the air in the exposure chamber was monitored throughout the exposure—continuous measurement. Animals from groups M, MEth, F, FEth were exposed to tobacco smoke at a concentration of 1500 mg CO/m^3^ of air. The exposure lasted for 5 days, 6 h a day. After exposure, rats were anesthetized by intramuscular administration of ketamine with xylazine at a dose of 200 mg ketamine/kg + 10 mg of xylazine/kg and blood was collected from the heart ventricle at six-time intervals (0.25, 0.5, 1, 2, 3, 5 h) with three rats per time points Nicotine and its metabolites: cotinine, trans-3'-hydroxycotinine, nornicotine, nicotine N’-oxide, and cotinine N-oxide, were determined in the collected plasma.

### Determination of ethyl alcohol

The method and results of the determination of ethanol, its major metabolite, and other alcohols were described previously^19,34^. The concentration of ethanol was determined by gas chromatography (ATI UNICAM 610 Series gas chromatograph equipped with a flame ionization detector) after headspace solid-phase microextraction (MSPE). First-order alcohol (isobutyl) was used as the internal standard. The limit of detection (LOD) was 2 mg/L and quantification (LOQ) 5 mg/L.

### Determination of nicotine, nornicotine, nicotine N-oxide, cotinine, trans-3'-hydroxycotinine, and cotinine N-oxide

NIC, NOR, NICNO, COT, 3HC, and COTNO in plasma (1 mL) were determined by high-performance liquid chromatography coupled with tandem mass spectrometry using deuterated cotinine as the internal standard, after prior liquid–liquid extraction (pH 8, NaOH, dichloromethane-n-propanol in ratio 9:1).

High-performance liquid chromatography was conducted using an Agilent 1200 RR with an Agilent 6410 Triple Quad mass detector as described previously^19^. Chromatographic separation has been achieved using a Merck^®^ RP-Select B HPLC column (105 mm × 4.6 mm, 5 µm) with a gradient system consisting of 10 mmol/L ammonium acetate (pH 5.5) (A), and methanol (B) at a flow rate of 1 mL/min. The injection volume was 25 µL. The gradient course was: minutes 0–14—linear growth from 5 to 100% of B solution, then reversal within the next 3 min and column conditioning in the next 5 min. The mass spectrometer was operated in electrospray positive mode using MRM data acquisition. The following ESI conditions were applied: gas temperature—350 °C, gas flow—12 L/min, nebulizer pressure: 50 psi, capillary voltage—4 kV. D_3_-cotinine was used as an internal standard. Retention times and MRM transitions for nicotine and its metabolites are presented in Table 1.

**Table 1:** Retention times, MRM transitions for nicotine and its metabolites.

Compound	t_R_ [min]	m/z precursor	m/z fragment 1	m/z fragment 2
Nicotine	9.8	163.1	130.1 (95/21)*	80.1 (95/25)
Nornicotine	6.6	149.1	132.1 (80/9)	80 (80/25)
Nicotine N-oxide	7.7	179.1	96 (80/21)	84.1 (80/17)
Cotinine	7.0	177.1	98 (110/21)	80 (110/25)
Trans-3'-hydroxycotinine	5.0	193.1	134 (110/17)	80.1 (110/33)
Cotinine N-oxide	3.7	193.1	98.1 (125/25)	96 (125/21)
D_3_-cotinine	7.0	180.1	101.1 (110/21)	80 (110/29)

*first value in parentheses fragment voltage [V], second value collision energy [V].

### Selected validation parameters

Before starting the tests, the method was validated by determining the detection limits, the limit of quantification, and repeatability during one day and between days. The limit of detection (LOD) and quantification (LOQ) were determined for various concentrations of cotinine in the plasma. An S/N (signal-to-noise) = 3 ratio was adopted as the limit of detection (LOD). The nicotine limit of detection was 2.29 ng/mL, and the limit of quantification was 6.86 ng/mL. The nornicotine LOD was 0.93 ng/mL and LOQ was 3.48 ng/mL. Nicotine N-oxide LOD was 1.35 ng/mL and the LOQ was 4.06 ng/mL. The cotinine LOD was 0.76 ng/mL and LOQ was 2.29 ng/mL. Trans-3'-hydroxycotinine LOD was 1.24 ng/mL and the LOQ was 3.72 ng/mL. The cotinine N-oxide LOD was 0.08 ng/mL and the LOQ was 0.25 ng/mL.

The linearity of the analytical method for NIC and COT ranged from the LOQ to 550 ng/mL; for 3HC, from the LOQ to 100 ng/mL; for NOR, from the LOQ to 50 ng/mL; for NICNO and COTNO, from the LOQ to 10 ng/mL.

The repeatability of the method was tested for two concentrations by analyzing each substance in a ten-element series. Plasma samples with a standard addition were tested on a given day and on different days. The obtained results are summarized in Table 2.

**Table 2:** Repeatability of the plasma nicotine and their metabolites determination of the day and between days.

Compound	Concentration [ng/mL]	CV [%]	
		In day	Between days
Nicotine	10	6.39	18.66
	300	10.50	10.99
Nornicotine	10	8.73	9.97
	300	10.04	10.35
Nicotine N-oxide	10	8.87	6.07
	300	5.00	8.48
Cotinine	10	7.44	4.87
	300	0.32	3.73
Trans-3'-hydroxycotinine	10	4.78	5.79
	300	2.72	3.55
Cotinine N-oxide	10	5.34	4.96
	300	3.53	10.52

### Statistical and pharmacokinetic analysis

Pharmacokinetic analysis of NIC, NOR, NICNO, COT, 3HC, and COTNO was carried out by the NCA—non-compartmental(al) analysis (statistical moment analysis) using the SPLINE computer program. The following parameters were calculated: area under the curve (AUC) and mean resident time (MRT).

$$AUC = \int\limits_{0}^{\infty } {C(t)dt} \Rightarrow \frac{1}{AUC}\int\limits_{0}^{\infty } {C(t)dt} = 1MRT = \frac{1}{AUC}\int\limits_{0}^{\infty } {C(t)tdt}$$

Analysis of variance was used with post-hoc Tukey HSD test to compare the calculated pharmacokinetic parameters. The data were considered statistically significant when p < 0.05. All statistical calculations were carried out with the STATISTICA 13.0 computer program (StatSoft, Poland).

## Results

There are no specific reports in the current literature about the effect of chronic alcohol consumption on the biotransformation of nicotine in rodent (rat) species, where the nicotine has been administrated via inhalation of tobacco smoke. In this study, plasma concentrations of nicotine and its metabolites were measured after five days of exposure of rats to tobacco smoke. From the previous our studies on the effects of exposure to tobacco smoke on the pharmacokinetics of ethanol^16^ we can conclude that exposure to tobacco smoke has an insignificant impact on the elimination of ethyl alcohol, causing a significant increase only in the volume of distribution, and tended to decrease the level of acetic aldehyde (main ethanol metabolite).

### Nicotine concentration

**Figure 3:**
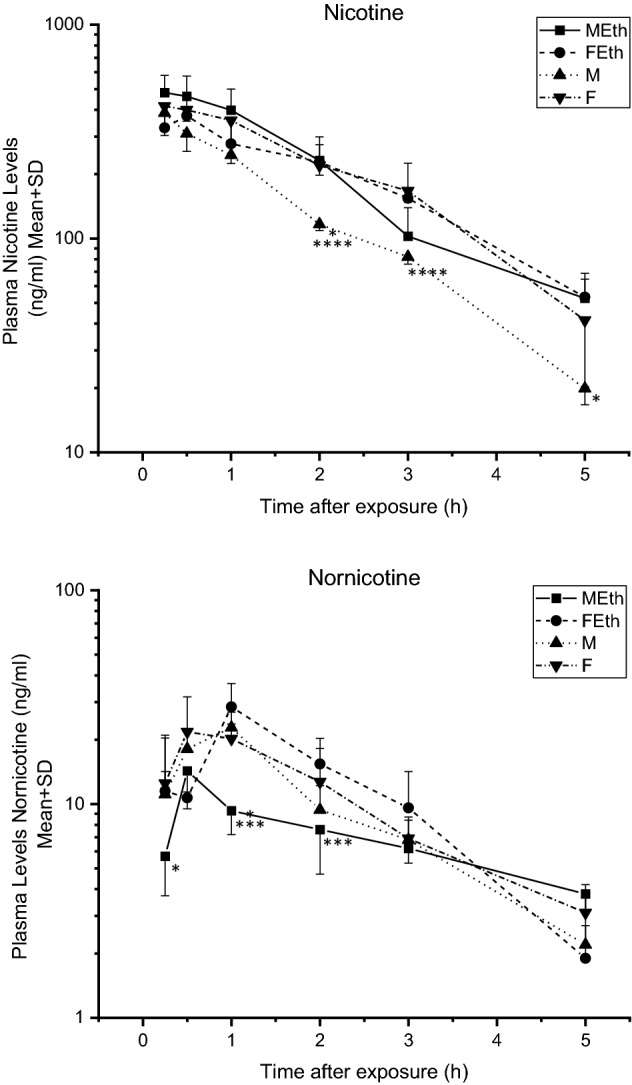
Plasma nicotine and nornicotine levels measured over time—rats exposed to tobacco smoke at a concentration, expressed as carbon monoxide, 1500 mg CO/m^3^ for 5 days, 6 h a day. Asterisks indicate a significant difference value (P < 0.05): *MEth vs M, **FEth vs F, ***MEth vs FEth and ****M vs F.

The profile of changes in the nicotine concentration in the plasma of rats that were ethanol-preferring, non-preferring, and exposed to tobacco smoke is presented in Fig. 3. The maximal concentration of NIC in the MEth group was achieved 0.25 h after the end of exposure (481.30 ng/mL). Similarly, in the M group (386.70 ng/mL) and F group (416.20 ng/mL), the maximum concentration was achieved 0.25 h after the end of exposure to tobacco smoke. In the FEth group, the maximal concentration was observed after 0.5 h (375.10 ng/mL). Plasma NIC levels were lower in the M group compared with the F group at 2 and 3 h, and compared with the MEth group at 2 and 5 h following acute nicotine administration (P < 0.05) (Fig. 3).

The calculated AUC in the case of the studied animal groups ranged from 707.00 ± 84.43 ng/mL/h (M group) to 1151.67 ± 223.68 ng/mL/h (F group) however, these differences were not statistically significant (Table 3). A significant difference (p < 0.05) was observed for the MRT of nicotine between male and female ethanol preferring rats, and between male and female non-ethanol- preferring rats.

**Table 3:** Pharmacokinetic parameters of plasma nicotine and metabolites in alcohol preferring and non-preferring rats.

Parameter	M	MEth	F	FEth
**Nicotine**				
AUC [ngh/mL]	707.00 ± 84.43	1109.63 ± 303.05	1151.67 ± 223.68	1094.83 ± 338.16
MRT [min]	1.77 ± 0.06^d^	2.02 ± 0.06^c^	2.27 ± 0.24	2.51 ± 0.22
**Nornicotine**				
AUC [ngh/mL]	51.19 ± 10.65	48.95 ± 9.25	59.53 ± 23.77	62.05 ± 20.47
MRT [min]	1.96 ± 0.19	4.11 ± 0.62^a,c^	2.41 ± 0.23	2.13 ± 0.19
**Nicotine-N-oxide**				
AUC [ngh/mL]	6.72 ± 1.61	5.93 ± 1.00	11.58 ± 5.49	2.35 ± 0.95^b^
MRT [min]	3.35 ± 0.09	4.29 ± 0.90	4.21 ± 0.71	3.10 ± 0.95
**Cotinine**				
AUC [ngh/mL]	2209.00 ± 517.60	4651.00 ± 960.64	3794.67 ± 1424.60	3274.67 ± 1535.59
MRT [min]	5.55 ± 1.81	16.54 ± 1.34^a,c^	7.72 ± 4.04	5.47 ± 2.62
**Trans-3’-hydroxycotinine**				
AUC [ngh/mL]	574.23 ± 151.25	403.05 ± 140.60	840.27 ± 159.49	442.27 ± 112.39^b^
MRT [min]	11.08 ± 0.31	13.16 ± 2.60^c^	11.62 ± 0.94	8.97 ± 1.41
**Cotinine-N oxide**				
AUC [ngh/mL]	24.51 ± 11.18	6.48 ± 2.37	20.01 ± 8.82	5.46 ± 1.41
MRT [min]	3.51 ± 0.07	4.58 ± 1.65	3.46 ± 0.31	3.24 ± 0.45

Data represent mean ± *SD*, *n* = 18 in each group. *AUC* total area under plasma concentration–time curve for the dosing interval, *MRT* mean residence time.

^a^Statistically significant differences between male rats preferring ethanol versus non-preferring ethanol.

^b^Statistically significant differences between female rats preferring ethanol versus non-preferring ethanol.

^c^Statistically significant differences between male rats preferring ethanol versus female rats preferring alcohol.

^d^Statistically significant differences between male rats non-preferring ethanol versus female rats non-preferring ethanol.

### Nornicotine concentration

Figure 3 shows the profile of changes in the nornicotine concentration in the plasma of rats that were ethanol preferring, non-preferring, and exposed to tobacco smoke. The highest plasma NOR concentrations peaked at 0.5 h in male alcohol-preferring rats (14.28 ng/mL) and at 1 h in M (22.80 ng/mL), FEth (28.50 ng/mL), and F (20.20 ng/mL). Plasma NOR levels were lower in the MEth group compared with the M group at 0.25 and 1 h and compared with the FEth group at 1 and 2 h following acute nicotine administration (P < 0.05) (Fig. 3). No statistical differences were observed for AUC between the studied groups (Table 3). The MRT was significantly higher in the MEth group in comparison with groups M and FEth.

### Nicotine N-oxide concentration

**Figure 4:**
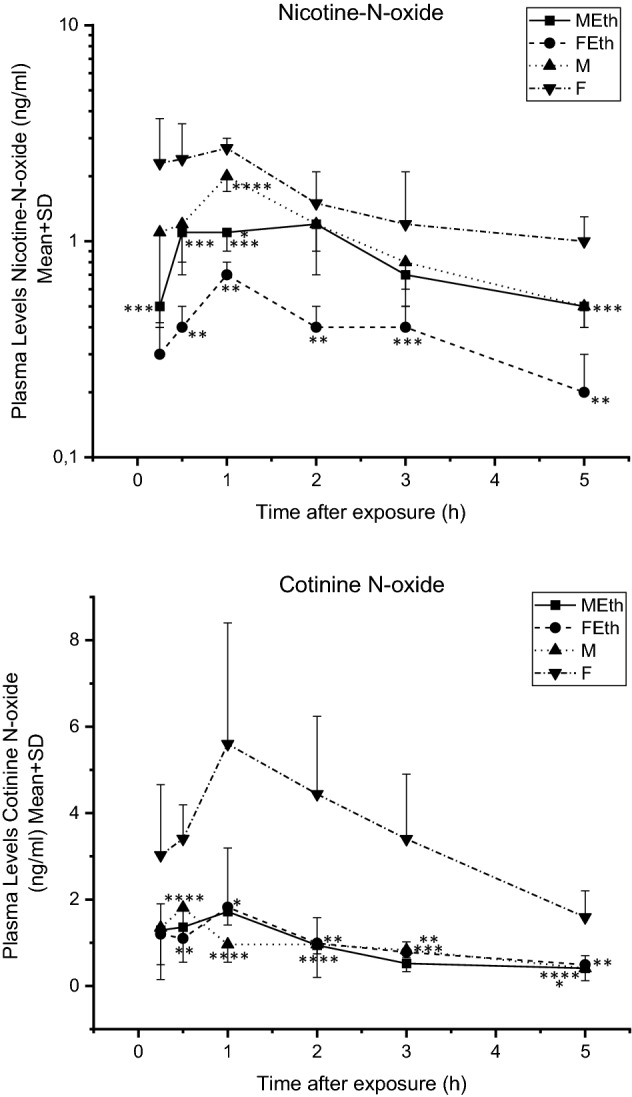
Plasma nicotine-N-oxide and cotinine-N-oxide levels measured over time—rats exposed to tobacco smoke at a concentration, expressed as carbon monoxide, 1500 mg CO/m^3^ for 5 days, 6 h a day. Asterisks indicate a significant difference value (P < 0.05): *MEth vs M, **FEth vs F, ***MEth vs FEth and ****M vs F.

Plasma concentrations of NICNO were as follows: at 2 h for MEth (1.25 ng/mL) and 1 h for M (2.04 ng/mL), FEth (0.73 ng/mL), and F (2.72 ng/mL) (Fig. 4) Plasma NICNO levels were lower in the MEth group compared with the FEth group at 0.25, 0.5, 1, 3, and 5 h and compared with the M group at 1 h. Moreover, lower plasma NICNO levels were noted in the FEth group compared with the F group at 0.5, 1, 2, and 5 h, and between the M group and the F group at 1 h following acute nicotine administration (P < 0.05) (Fig. 4). The calculated area under the curve for nicotine N-oxide concentration–time in the case of animals from the F group amounted to 11.58 ± 5.49 ng/mL/h and was higher than in the case of animals from the FEth group (2.35 ± 0.95 ng/mL/h), and these differences were statistically significant (Table 3). Statistically significant differences were not observed for the MRT parameter.

### Cotinine concentration

**Figure 5:**
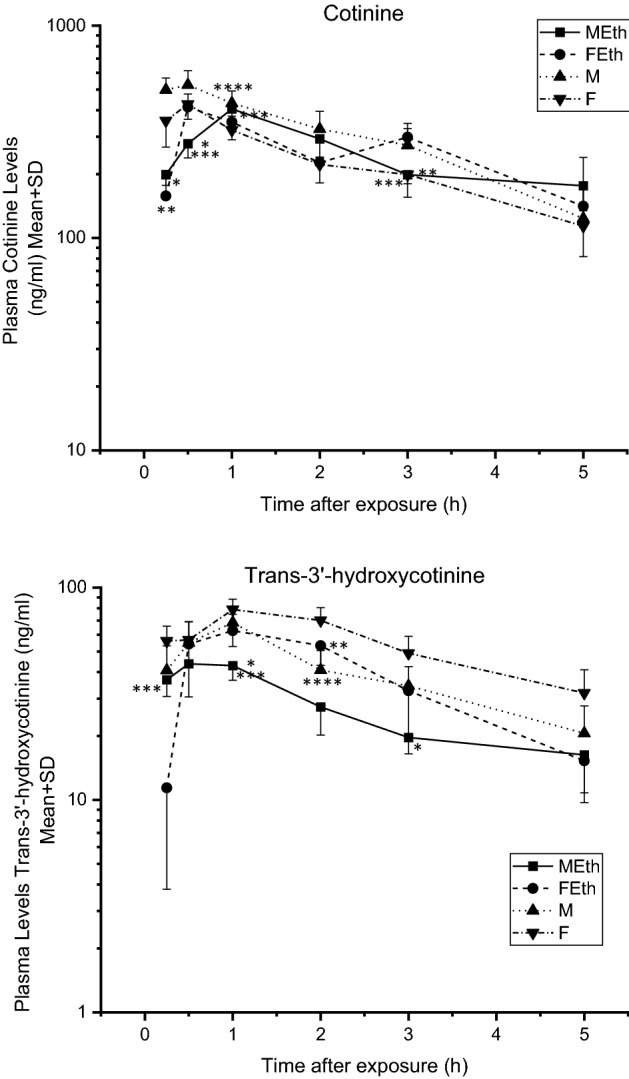
Plasma cotinine and trans-3’-hydroxycotinine levels measured over time—rats exposed to tobacco smoke at a concentration, expressed as carbon monoxide, 1500 mg CO/m^3^ for 5 days, 6 h a day. Asterisks indicate a significant difference value (P < 0.05): *MEth vs M, **FEth vs F, ***MEth vs Feth and ****M vs F.

The maximum cotinine concertation in plasma in the MEth group was 405.40 ng/mL and was observed 1 h after exposure to tobacco smoke (Fig. 5). Plasma cotinine concentrations peaked after 0.5 h in groups M (526.10 ng/mL), FEth (413.91 ng/mL) and F (426.70 ng/mL).

Plasma COT levels were lower in the MEth group compared with the M group at 0.25 and 0.5 h and compared with the FEth group at 0.5, 1, and 3 h. Moreover, lower plasma COT levels were noted in the FEth group compared with the F group at 0.25 h following acute nicotine administration (P < 0.05). Higher plasma cotinine levels were noted after 3 h in the FEth group compared with the F group. Plasma COT levels were lower in the F group compared with the M group at 1 h (Fig. 5). Statistical differences were observed for cotinine MRT between male rats preferring (16.54 ± 1.34 h) versus non-preferring ethanol (5.55 ± 1.81 h), and between male versus female rats preferring alcohol (5.47 ± 2.62 h) (Table 3).

### Trans-3'hydroxycotinine concentration

Figure 5 shows the profile of changes in trans-3'hydroxycotinine concentration in the plasma of animals that were ethanol-preferring, non-preferring and exposed to tobacco smoke. The highest plasma trans3'hydroxycotinine concentrations peaked at 0.5 h in group MEth (43.80 ng/mL), and 1 h in M (68.40 ng/mL), FEth (62.70 ng/mL) and F (78.90 ng/mL).

Plasma 3HC levels were lower in the MEth group compared with the FEth group at 1 h and higher compared with the FEth group at 0.25 h. Moreover, lower plasma 3HC levels were noticed in the MEth group compared with the M group at 1 and 3 h following acute nicotine administration (P < 0.05). Lower plasma 3HC levels were noted after 2 h in the FEth and M groups compared with the F group (Fig. 5).

The area under the concentration–time curve was similar in the three groups, ranging from 403.05 ± 140.60 ng/mL/h to 574.23 ± 151.25 ng/mL/h. A statistical difference was observed for AUC between ethanol-preferring and non-preferring female rats (442.27 ± 112.39 ng/mL/h; 840.27 ± 159.49 ng/mL/h). MRT was significantly higher in the MEth in comparison with the Feth group (Table 3).

### Cotinine N-oxide concentration

Plasma concentrations of COTNO peaked the fastest for M, after 0.5 h (1.81 ng/mL). The highest plasma cotinine N-oxide concentration was observed after 1 h for MEth (1.72 ng/mL), FEth (1.82 ng/mL), and F (5.60 ng/mL) (Fig. 4). Plasma COTNO levels were lower in the FEth group compared with the F group at 0.5, 2, 3, and 5 h. Moreover, lower plasma COTNO levels were noted in the M group compared with the F group at 0.5, 1, 2, 3, and 5 h following acute nicotine administration (P < 0.05). Higher plasma COTNO levels were noted after 1 h in the MEth group compared with the M group, and lower levels in the MEth group compared with the M group at 5 h (Fig. 4).

Statistically significant differences were not observed for the AUC and MRT parameters. The calculated area under the curve for cotinine N-oxide concentration—time and MRT in the case of the studied animal groups amounted to from 5.46 ± 1.41 ng/mL/h to 24.51 ± 11.18 ng/mL/h for AUC, and from 3.24 ± 0.45 h to 4.58 ± 1.65 h for MRT, but these differences were not statistically significant (Table 3).

## Discussion

Nicotine is the main addictive and toxic component of cigarette smoke, in terms of risk of many diseases, including coronary heart disease, chronic obstructive pulmonary disease, and many cancers e.g. lungs, and bladder^4^. Inhalation research in rodents is well established, widespread, and used to study nicotine dependence and its toxicity^3,4,19,35–37^.

In this study, a sensitive, precise, and accurate LC–MS/MS method was used to quantify nicotine and five major metabolites in the plasma of ethanol-preferring and non-ethanol preferring (male and female) rats exposed to tobacco smoke. The conducted study indicates rapid absorption, and slow elimination of nicotine in rats.

In most cases, the elimination of nicotine in mammals is via conversion to cotinine by C-oxidation. The oxidative metabolism of nicotine also produces a lower amount of nicotine metabolites such as nornicotine and nicotine N-oxide^38^. Both in humans and rats, nicotine is metabolized to nornicotine by N-demethylation^38,39^.

The pharmacokinetic (model-independent pharmacokinetics) parameters of nicotine and its metabolites calculated on the basis of plasma concentrations in rats are presented in Table 3. As shown in Figs. 3, 4, 5, the concentration–time profiles of nicotine metabolites were clearly biphasic. Based on the obtained results, we can conclude that FEth eliminate nicotine faster than MEth. Moreover, female non ethanol-preferring rats eliminate nicotinefaster than male non-ethanol-preferring rats. Sex hormones can affect nicotine metabolism. In a study by Benowitz et al., it was found that nicotine is metabolized faster in women than in men^5^ a similar phenomenon was observed in our studies in rats. Metabolic tolerance to nicotine may be increased by inhalation than by other routes of administration^40^. Nicotine is distributed predominantly in the lean mass therefore a difference in body weight composition could potentially affect the volume of distribution of NIC^10^. The female rats used in our experiment had almost two times less body weight than males, which is in line with the physiological data of these animals. Alcohol preferring male rats eliminate nornicotine faster than alcohol-preferring female rats and non-alcohol-preferring male rats. In our research, this relationship is not unequivocal. The F-group showed higher exposure to nicotine N-oxide than the FEth-group. In our opinion, alcohol (not supported by enzyme tests) may reduce the activity of nicotine N-oxide metabolizing enzymes. Alcohol-preferring male rats eliminate cotinine faster than non-alcohol-preferring male rats and alcohol preferring female rats. Cotinine metabolism increases as the level of nicotine in the blood decrease. Absorbed nicotine is rapidly and extensively metabolized in the liver to inactive cotinine. The rate of COT elimination is 2–8 times higher than that of NIC. Cotinine is a small and polar compound that easily crosses cell membranes. The distribution of COT from the circulatory system into the peripheral tissues is fast and thorough. The elimination of COT from the circulatory system is rapid due to its high water solubility and membrane permeability. The results of this study revealed an important pharmacokinetic parameter of COT, namely a faster elimination dependent on alcohol preference. Female non-alcohol-preferring rats were more exposed to trans3-'hydroxycotinine than female alcohol-preferring rats. On the other hand male rats preferring alcohol eliminate trans3'hydroxycotinine faster than female rats preferring alcohol. The higher the 3HC/COT ratio, the greater the activity of CYP2A6 and the faster the rate of nicotine metabolism.

The rapid absorption of NIC and intense metabolism leads to its rapid disappearance from the plasma. Nicotine first activates and then decreases the sensitivity of the nicotinic dopamine receptors of the midbrain, and also enhances glutamatergic stimulation, and reduces GABAergic inhibition of these neurons, resulting in prolonged enhanced effects of these neuromediators^41^. It should be emphasized that the acute and chronic effects of nicotine are different, the chronic effects of nicotine differ in females as opposed to males and depend on the dose of nicotine and age. There are gender differences in susceptibility to nicotine and alcohol. Women become addicted to nicotine or alcohol more often than men^42,43^. In men, nicotine increases choline acetyltransferase, while ethanol reverses this effect. In women, nicotine reduces this enzyme. The GABA-ergic and glutamatergic effects of alcohol are region-specific and sex-selective, which are influenced by sex differences in the composition of the respective receptor subunits^41^.

The interaction between chronic alcohol abuse and smoking may have an effect on the antioxidant defense system and lipid peroxidation in specific rat tissues^44^. Marselos et al. reported that the effects of nicotine on the body may affect the activity of certain enzymes in the liver and the metabolism of ethanol in rats^45^. Loscutoff et al. state that there is a faster exchange of inhaled gases with pulmonary capillary blood in rats as compared to humans as a result of more rapid circulatory and respiratory rates^46^. This exchange may lead to higher blood levels of carboxyhemoglobin in rats. It has been documented that short-term and long-term exposures often produce different results^47^.

According to Luo et al., chronic administration of ethanol reduced the half-life of nicotine in mice by approximately 50%. Furthermore, consumption of ethanol altered certain aspects of nicotine pharmacokinetics; blood levels of nicotine were lower in mice given long-term drinking ethanol^48^. Our research has shown that the FEth group has lower blood levels of nicotine than the F group in each measuring point. Male rats metabolize nicotine faster than female rats. According to Kyerematen et al., the castration of male rats reduces nicotine metabolism, and the castration effect is reversed by the administration of testosterone^6^. Nicotine causes the release of many types of neurotransmitters, neuropeptides and steroid hormones, and administration of high doses of nicotine causes toxic effects orstress. These complex effects of nicotine may have hampered progress in characterizing the regulation of nicotine metabolism^20^.

In studies conducted by Ignatowicz et al.^3^, it was noticed that combined exposure to tobacco smoke and alcohol caused greater damage to the DNA of the liver and lungs than measured after a single dose of alcohol or exposure to tobacco smoke alone. Values obtained in the lungs of rats treated with alcohol or tobacco smoke with alcohol were significantly greater than the corresponding values in the liver, suggesting that the lungs are more sensitive to exogenous oxidants. In ethanol-dependent rats, the combined exposure to smoke and alcohol differently modulates the endogenous antioxidant defense system and responses to oxidative stress^3^.

To sum up, chronic consumption of large amounts of alcohol may lead to an increase in the rate of biotransformation of nicotine and its metabolites and affect its distribution kinetics in a gender-specific manner. Numerous studies have shown that the potential effect of ethanol on the volume of distribution of nicotine can be explained by the influence of ethanol on the structure and permeability of the cell membrane as well as on the function of the epithelial barrier^49^. Ethanol also influences nicotine clearance, possibly by increasing renal clearance or the efficacy of in vivo hepatic metabolic clearance. Many studies show that smokers who consume alcohol regularly may have lower levels of nicotine in their urine compared with non-drinking smokers. Chronic exposure to nicotine and ethanol, alone or in combination, may modify the pharmacokinetics of nicotine in vivo in humans, as seen in rats.
